# Positive taxis and sustained responsiveness to water motions in larval zebrafish

**DOI:** 10.3389/fncir.2015.00009

**Published:** 2015-03-06

**Authors:** Antonia H. Groneberg, Ulrich Herget, Soojin Ryu, Rodrigo J. De Marco

**Affiliations:** Developmental Genetics of the Nervous System, Max Planck Institute for Medical ResearchHeidelberg, Germany

**Keywords:** larval zebrafish, behavior, hydrodynamic sensing, lateral line, sensory responsiveness

## Abstract

Larval zebrafish (*Danio rerio*) have become favored subjects for studying the neural bases of behavior. Here, we report a highly stereotyped response of zebrafish larvae to hydrodynamic stimuli. It involves positive taxis, motion damping and sustained responsiveness to flows derived from local, non-stressful water motions. The response depends on the lateral line and has a high sensitivity to stimulus frequency and strength, sensory background and rearing conditions—also encompassing increased threshold levels of response to parallel input. The results show that zebrafish larvae can use near-field detection to locate sources of minute water motions, and offer a unique handle for analyses of hydrodynamic sensing, sensory responsiveness and arousal with accurate control of stimulus properties.

## Introduction

Due to their genetic amenability and transparent body, larval zebrafish have become favored subjects for studying the neural bases of behavior. Substantial advances in optogenetics and high-resolution *in vivo* imaging have been made (Friedrich et al., 2010; Renninger and Orger, 2013), and neural activity has been successfully correlated to eye and tail movements in zebrafish larvae exposed to whole-field visual stimuli (Ahrens et al., 2013; Kubo et al., 2014; Portugues et al., 2014; Severi et al., 2014). Despite advancements in techniques for measuring neural activity, the exploration of goal-directed actions appropriate for synchronized measurements of brain activity and behavior has been lagging behind, particularly as compared to research in rodents. While locomotor patterns and swim kinematics have been examined in detail, the repertoire of goal-directed behaviors in larval zebrafish (Fero et al., 2011) has yet to be explored in full. Here, we searched for a goal-directed response of zebrafish larvae to non-stressful water motions.

The detection of underwater motion and pressure waves is widespread in the animal kingdom, and provides many species of fish with varied benefits ranging from object detection to sensing conspecifics (Bleckmann, 1986; Hawkins, 1986; Kalmijn, 1988). Water motions can have abiotic sources, such as currents or stationary objects that distort self-generated flows, or biotic sources, such as prey and predator motions or conspecific vocalizations. Hydrodynamic sensing is thus thought to contribute to various responses and behavioral categories, including feeding, predator avoidance, orientation and intraspecific communication (Montgomery et al., 2014). Fish evolved dedicated sensors and brain circuits to detect hydrodynamic fields and acoustic cues. They sense flows and sounds by the lateral line, sensitive to current (Hofer, 1908; Dijkgraaf, 1963; Bleckmann, 1986; van Netten and McHenry, 2014), and the inner ear, sensitive to linear acceleration and gravity (Hawkins, 1986). In the zebrafish larva, the inner ear and lateral line develop within 1 week post fertilization, including both afferent and efferent connections (Metcalfe et al., 1985; Haddon and Lewis, 1996).

Using computer-vision-based methods, we found that larval zebrafish can approach sources of non-stressful water flows, and that these flows can elicit, if repeated, a graded, stimulus-frequency-dependent response. When presented with local water motions, larvae can move quickly towards the source of the ensuing hydrodynamic fields. If evoked at 1–5 Hz, these fields first cause them to reduce their overall locomotion gradually, and, then, to remain virtually motionless in the proximity of the source as long as the stimuli persist—once minimum locomotion is reached, threshold levels of response to parallel input increase. After the offset of the stimuli, regular locomotion is re-established only gradually. The motor response is highly stereotyped and remains stable over multiple stimulations. Its magnitude depends critically on distinctive stimulus properties, sensory background and rearing conditions. Chemical ablation of sensory cells reveals that the response depends on the integrity of the lateral line, leading the way to detailed analyses of the neural circuits involved. The results demonstrate that zebrafish larvae can use near-field detection to locate sources of water motions. Altogether, they add a robust phenotype to a growing repertoire of laboratory behaviors and provide an interesting opportunity for analyses of hydrodynamic sensing, sensory responsiveness and arousal with accurate control of stimulus properties.

## Materials and Methods

### Animal Husbandry and Handling

Zebrafish breeding and maintenance was performed under standard conditions (Westerfield, 2000). Embryos were collected in the morning and raised on a 12:12 light/dark cycle in E2 medium at 28°C (Westerfield, 2000). All experiments were carried out with wild-type zebrafish (cross between AB and TL strains) at 6 days post fertilization (dpf), unless otherwise stated. Tests were conducted between 9:00 and 18:00, with different experimental groups intermixed throughout the day. To raise larvae either in isolation or in groups, eggs were collected and placed in plastic dishes (internal diameter: 35 mm) either individually or in groups of 20, respectively. Zebrafish experimental procedures were performed according to the guidelines of the national animal welfare law and approved by the local government.

### Setup

**Figure 1:**
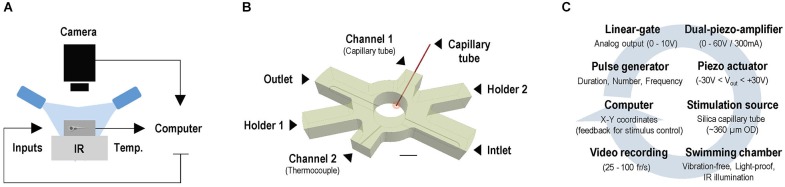
**Setup for video tracking zebrafish larvae in combination with water motions. (A)** Configuration for imaging freely swimming larvae under infrared illumination and constant temperature, in combination with locally evoked water motions. **(B)** Swimming chamber with water inlet and outlet and two side channels. Side channels allow passage of a thermocouple and a rigid silica capillary tube, with one end submerged into the medium (stimulus source, red circle) and the other fixed to a bender actuator causing lateral displacements (LDs) of the submerged tip. Scale bar, 5 mm. **(C)** A computer-controlled pulse generator triggers unidirectional LDs of controllable speed via a dual piezo amplifier and a TTL control system; LDs can be triggered according to a larva’s position within the chamber via online video tracking.

Experiments were conducted under infrared (IR) light delivered through a custom-made array of IR-LEDs mounted inside a light-proof enclosure. Larvae were imaged through infrared-sensitive cameras, at either 25 (ICD-49E B/W, Ikegami Tsushinki Co., Ltd. Japan) or 100 frames*s^−1^ (Firewire Camera, Noldus Information Technology, Wageningen, Netherlands), with a lens (TV Lens, Computer VARI FOCAL H3Z4512 CS-IR, CBC; Commak, NY, USA) positioned above a cylindrical custom-made swimming chamber (internal diameter: 10 mm, height: 10 mm) holding a volume of 400 μl of E2 medium (Figure 1A). Motion values from video recordings made at 25 and 100 frames*s^−1^ are expressed as distance swum every 40 (mm*(40 ms)^−1^) and 10 ms (mm*(10 ms)^−1^), respectively. The complete setup was placed on a vibration-free platform (Newport Corp., Irvine, CA, USA). We used EthoVision XT software (Noldus Information Technology, Wageningen, Netherlands) and algorithms written in MATLAB 2009b (MathWorks, Inc., Natick, MA, USA) to monitor the movements of larvae swimming either individually or in groups. The swimming chamber (Figure 1B) had two cylindrical channels (internal diameter: 400 μm), with their longest axis oriented at an angle of 30° relative to horizontal. They were symmetrically situated at opposite sides of the chamber and opened 200 μm above its transparent glass bottom. One of these two side channels allowed passage of a segment of a rigid silica capillary tube (outer diameter: 350 μm, full length: 25 mm). One end of the capillary tube (henceforth: stimulus source) was submerged (~400 μm) into the chamber’s inner medium (depth: 5 mm), and the other fixed to a multilayer bender actuator (PICMA® PL140.10, Physik Instrumente (PI) GmbH + Co. KG, Karlsruhe, Germany) with an operating voltage of 0–60 V, a maximum displacement of ±1000 μm and an unloaded resonant frequency of 160 Hz. The bender, coupled to a pulse generator, a dual piezo amplifier and a TTL control system, produced unidirectional lateral displacements (henceforth: LDs) of the capillary’s submerged tip, of 50 μm and controllable speed, creating minute flows within the chamber. The input voltage applied to the actuator determined the speed of the capillary’s LDs (Figure 1C). The second side channel held a thermocouple (TS200, npi electronics GmbH, Tamm, Germany) that monitored the temperature inside the chamber (Figures 1A,B) and provided feedback to a control system (PTC 20, npi electronics GmbH, Tamm, Germany; Exos-2 V2 liquid cooling system, Koolance, Auburn, WA, USA) that kept the inner medium at 28°C (±0.1°C). In experiments with flowing water, a peristaltic pump (IPC Ismatec, IDEX Health and Science GmbH, Wertheim, Germany) and two opposite overtures at the bottom of the chamber (inlet and outlet, width: 2.5 mm, height: 400 μm, oriented at 90° relative to the longest axes of the side channels) allowed E2 medium (kept at 28°C ± 0.1°C) to flow at a constant flow rate of 200 μl*min^−1^. In all experiments, larvae moved freely either individually or in groups of eight, and were given an initial time period of 5 minutes to adapt to the chamber’s conditions prior to testing.

### Single LDs and Angle Measurements

**Figure 2:**
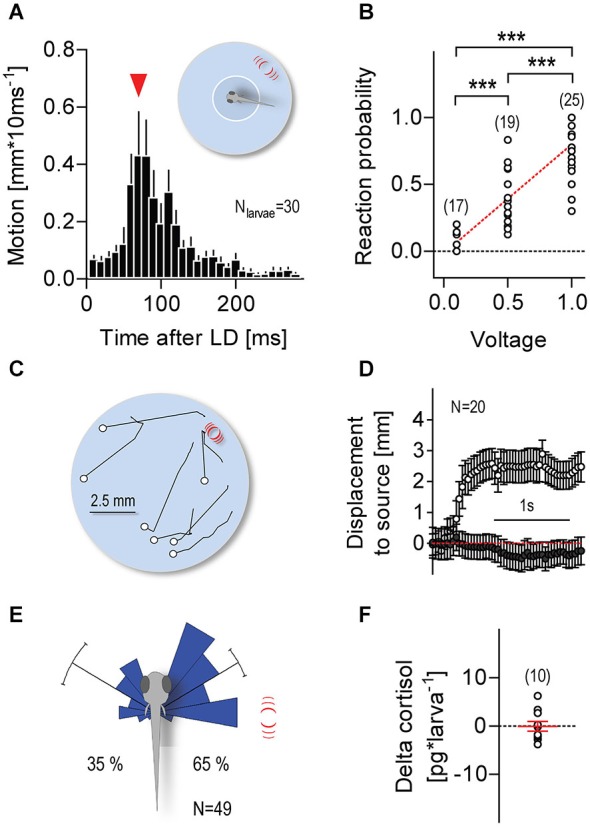
**Minute water motions elicit approach reactions. (A)** Distance swum every 10 ms by individual zebrafish larvae (mean ± S.E.M.) as a function of time after a single LD (pulse duration: 1 ms, input voltage: 1 V). LD onset occurs when a freely swimming larva enters a virtual circle at the center of the swimming chamber. Inset: white circle and red lines denote the virtual circle and the position of the stimulus source, respectively, not drawn to scale. Red arrowhead: latency until maximum distance swum in 10 ms. **(B)** Reaction probability as function of stimulus strength. Asterisks denote differences as determined by One-Way ANOVA, followed by *post hoc* comparisons (*p* < 0.001). **(C)** Exemplary 1 s motion traces from individual larvae after a single 1 ms LD (input voltage: 1 V). White circles and red lines denote the positions of the larvae at LD onset and the stimulus source, respectively. Larvae were video recorded at 25 frames*s^−1^ and LDs were evoked irrespective of their locations within the chamber. **(D)** Distance swum every 40 ms towards the stimulus source (mean ± S.E.M.) as a function of time. Gray and white circles denote measurements made before and after LD onset, respectively. **(E)** Direction of movement relative to body axis (0°) upon a 1 ms LD. Movements towards and away from the stimulus source shown as clockwise (<180°) and counter clockwise (>180) turns, respectively. Stimulus source (red lines) at 60–120°. See also Section Materials and Methods. **(F)** Whole-body cortisol of larvae exposed to consecutive LDs for 240 s, relative to untreated animals. Sample size in parentheses **(B,F)**.

Larvae were video recorded individually at 100 Hz; each video recording lasted 300 s. Single LDs (duration: 1 ms, input voltage: 1 V, unless otherwise indicated in the figures) were elicited only when the video tracking software detected the x-y coordinates of a larva’s head within a virtual circle (diameter: 3.5 mm) at the center of the swimming chamber (Figure 2A, insert). Over 300 s, freely swimming larvae elicited an average of 8.2 (±0.9) LDs, with an average time interval of 21.2 s (±2.1 s) between consecutive LDs. For each larva, we calculated “reaction probability” as the ratio between “the number of reactions over multiple LDs” and “the total number of LDs elicited over 300 s” (0.1 V: 6.1 ± 2.8, 0.5 V: 8.5 ± 3.6, 1 V: 8.2 ± 3.8), with reactions being defined as displacements larger than 0.5 mm * (10 ms)^−1^ occurring within 300 ms after LD onset. We used ImageJ (Freeware) to measure the directions of the larvae’s post-LD displacements. First, at the time of LD onset, we measured the angle formed between a larva’s body axis and a line connecting the center of its head and the center point of the capillary’s submerged end. Next, using the first 100 post-LD image frames (taken every 10 ms), we measured the distance moved and the angle formed by the larva’s body axis and the line connecting the start and end points of its post-LD displacement. In doing this, we used data only from LD presentations in which larvae had, upon entering the virtual space (i.e., at the time of LD onset), remained oriented at angles between 60° and 120° relative to the stimulus source.

### Cortisol Measurements

Groups of thirty 6 dpf larvae (experimental unit) were either exposed to LDs for 240 s (stimulated) or handled equally in the absence of LDs (control larvae). They were immobilized in ice water and collected 120 s after the offset of LDs. Samples were then frozen until subsequent cortisol extraction. Cortisol detection was carried out using a home-made cortisol ELISA protocol, as described in Yeh et al. (2013).

### Transient NaCl Exposure

Using a computer-controlled perfusion system (Octaflow, ALA Scientific Instruments, Inc., Farmingdale, NY, USA), 2 μl of NaCl solution of two different concentrations, either 2 or 5 M, were injected into a mixing compartment positioned 10 mm from the inlet of the swimming chamber. Teflon tubes (internal diameter: 230 μm, outer diameter: 600 μm) connected reservoirs of NaCl solution (combined with solenoid valves) with the mixing compartment and led the NaCl solutions to be mixed with the flowing E2 medium (flow: 200 μl*min^−1^) before reaching the chamber; TTL signals triggered the opening and closing of the valves (opening time: 1 s, pressure: 1 psi).

### Light Stimulation

A custom-made ring of LEDs surrounding the lens of the camera was positioned at a fixed distance above the swimming chamber (Figure 5C). The incident angle of the LEDs allowed for homogeneous illumination of the chamber’s inner compartment. Custom-made drivers, pulse generators and a TTL control box (USB-IO box, Noldus Information Technology, Wageningen, Netherlands) allowed computer control of the LEDs. Single freely swimming larvae were exposed to a 5 s square pulse of flashing blue light. The light pulse consisted of 100 ms flashes delivered at 5 Hz. Light power (0.8 mW*cm^−2^) was measured through a hand-held light power meter (Newport Corp., Irvine, CA, USA).

**Figure 3:**
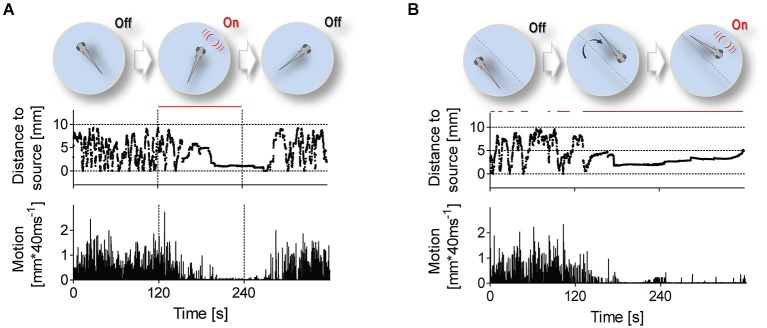
**Stimulation protocols**. Stimulation protocols (top) and exemplary traces of distance to stimulus source (middle) and swimming speed (bottom) from single 6 dpf larvae. Red lines depict time intervals when LDs occur at 1 Hz. **(A)** LDs can occur either uninterruptedly for 120 s, irrespective of a larva’s position within the swimming chamber, or **(B)** only when the larva swims near the stimulus source.

**Figure 4:**
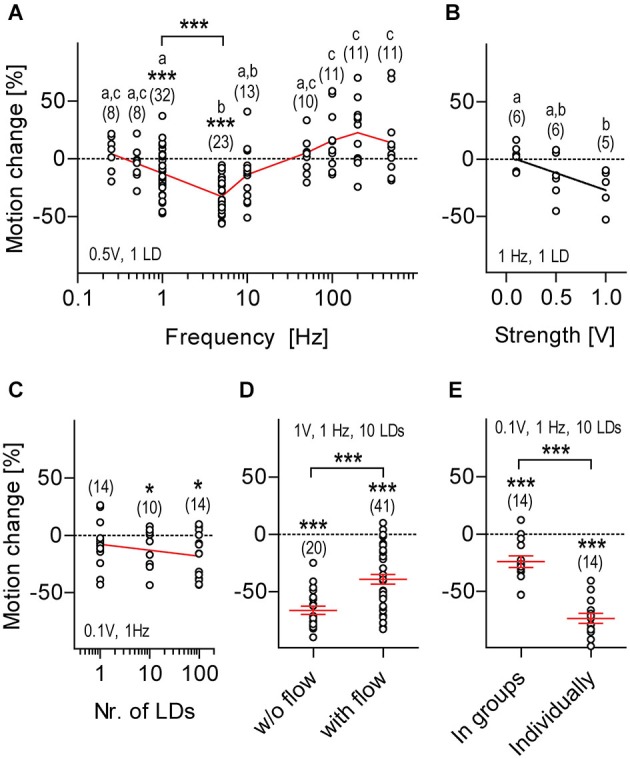
**Motion damping depends on distinctive stimulus properties, sensory background and rearing conditions**. LD-mediated locomotion relative to pre-stimulation level (henceforth: motion change, in %, all replicates) calculated over two consecutive 30 s periods (without and with LDs) as a function of LD frequency **(A)**, stimulus strength **(B)**, number of LDs per train **(C)**, sensory background **(D)** and rearing conditions **(E)**. Shown are LD variables and sample size (in parentheses), **(A,C–E)**. Asterisks above data points indicate results from One sample *t*-tests against 0 motion change. Asterisks above bars indicate results from Two-tailed *t*-tests, *p < 0.05, ***p < 0.001. Letters above sample size indicate results from *post hoc* comparisons after One-Way ANOVA, *p* < 0.05, **(A,B)**. Black line corresponds to linear regression, **(B)**. Red line connects means, **(A,C)**. Red lines and error bars depict means ± S.E.M. **(D,E)**.

**Figure 5:**
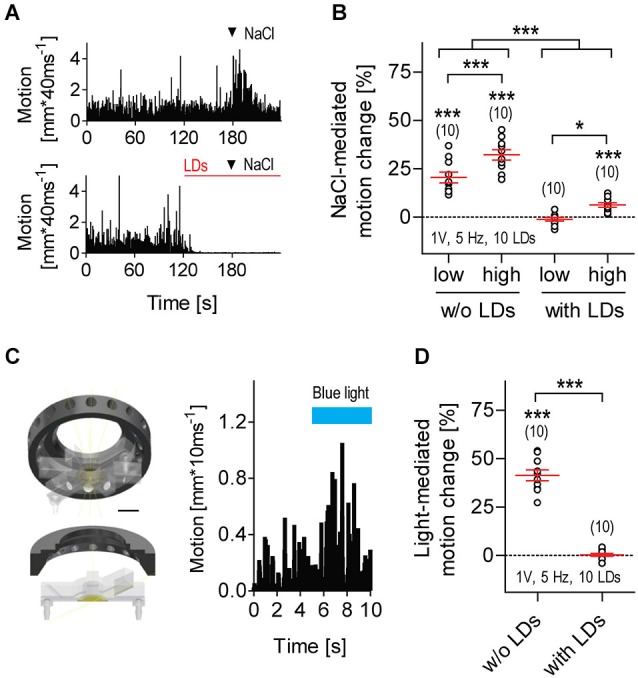
**LD-borne flows increase threshold levels of response to parallel input. (A)** Exemplary motion traces from larvae exposed to transient hyperosmolarity in the absence (top) or presence (bottom) of LDs (red line); arrow heads indicate addition of 2 μl NaCl_2M_ into the flowing E2 medium. **(B)** NaCl-mediated motion change relative to pre-stimulation baseline motion (in %, mean ± S.E.M.) in the absence or presence of LDs. NaCl_low_: 2 μl NaCl_2M_, NaCl_high_: 2 μl NaCl_5M_. Motion change as calculated for all conditions, over two consecutive 30 s periods, before and after addition of NaCl. Video recordings made at 25 frames*s^−1^. **(C)** Left: custom-made LED ring for blue light illumination, in combination with LD stimulation. The LED ring is positioned above the swimming chamber surrounding the lens of the camera, and provides homogeneous illumination of the inner medium. Scale bar, 10 mm. Right: exemplary motion trace from a larva exposed to a 5 s square pulse of flashing blue light. Flash duration: 100 ms, flash frequency: 5 Hz, light power: 0.8 mW*cm^−2^. **(D)** Light-mediated motion change relative to pre-stimulation level (in %, all replicates), in the absence or presence of LDs. Motion change as calculated over two consecutive 5 s periods, before and after light stimulation. Video recordings made at 100 frames*s^−1^. Shown are sample size (in parentheses) and LD variables. Asterisks over data points indicate results from One sample *t*-tests, **(B)**, and Wilcoxon Signed Rank tests, **(D)**, against 0 motion change. Asterisks over bars indicate results from *post hoc* comparisons after a Two-Way ANOVA, *p < 0.05, ***p < 0.001, **(B)**, and a Mann-Whitney test, **(D)**.

### Group Motion

To measure group motion, we used an algorithm in MATLAB 2009b (MathWorks, Inc., Natick, MA, USA) that detects movements of swimming larvae using the pixel-by-pixel mean squared error (*m.s.e*.) of transformed and adjusted images from consecutive video frames (De Marco et al., 2014), given by:

(1) $$m.s.e=\frac{1}{N}\sum_{pixel=1}^{N}{\left(image_{frame,pixel}-image_{frame-1,pixel}\right)}^{2}$$

where *N* corresponds to the total number of pixels of each frame. We confirmed that the *m.s.e*. remained constant in empty chambers, and that *m.s.e*. changes were exclusively due to the movements of swimming larvae. Motion change (with and without stimulation) was calculated in percentage relative to *m.s.e*. values from measurements during basal locomotion.

### Hair Cell Ablation

Several agents for pharmacological blocking of the lateral line are available (Coffin et al., 2014). We used copper sulfate, a previously described ototoxic agent (Hernández et al., 2006; Olivari et al., 2008). Larvae (5 dpf) were incubated overnight in either 0.1 or 1 μM CuSO_4_ solution in E2 medium and washed three times before behavioral testing or imaging. To visualize neuromasts, CuSO_4_-treated and untreated larvae were incubated in 1:100 NeuroTrace (green fluorescent Nissl stain, Molecular Probes, Life Technologies, Corp., Carlsbad, CA, USA) with 10% DMSO (Sigma-Aldrich Co. LLC, St. Luis, MO, USA) for 1 h at 28°C. Confocal images of intact, anesthetized larvae were taken in 1% Agarose Low Melt (Carl Roth GmbH + Co. KG, Karlsruhe, Germany) using a Leica SP5 CLSM with a 20× water objective (Leica Microsystems GmbH, Wetzlar, Germany). Confocal stacks were evaluated using Amira 5.4 (FEI Visualization Sciences Group, Burlington, MA, USA) to count hair cells by manual labeling in the segmentation editor.

### Statistical Analysis

All data are shown as single measurement points, mean and standard error of the mean (S.E.M.) or box-and-whisker plots. We used a random experimental design, Student’s *t*-tests (two-tailed) for two-group comparisons and ANOVAs for multiple group comparisons (followed by Bonferroni’s *post hoc* tests), or their non-parametric equivalents. We also used linear regression analysis. Analyses were carried out using MS-Excel (Microsoft Corp.; Redmond, WA, USA), Matlab 2009b (MathWorks, Inc., Natick, MA, USA), Prism 5 (Graphpad Software Inc., San Diego, CA, USA), Sigma Plot (Systat Software Inc., San Jose, CA, USA), ImageJ (Freeware), Oriana 4.0 (Kovach, Inc., Chandler, AZ, USA), R and VirtualDub (Freeware).

## Results

### Minute Water Motions Can Elicit Approach Reactions

Single larvae were presented with one or more LDs only if and as soon as they entered a small virtual circle at the center of the swimming chamber (Figure 2A, insert). Larvae entering the circle for the first time reacted to a single LD with a brief motion increase (maximum displacement: 1.3 ± 0.1 mm * (10 ms)^−1^) 90.0 ± 6.3 ms after LD onset (Figure 2A), a latency longer than those of short- (5.3 ms) and long-latency (28.2 ms) C-start reactions (Burgess and Granato, 2007). Larvae could then swim for a number of seconds and be presented with a new LD as soon as they re-entered the circle (see Section Materials and Methods). With long and varying inter-LD intervals, both reaction latency and maximum displacement remained invariant to the number of LD presentations (Kruskal-Wallis test, latency: *H* = 22.95, *p* = 0.12, max. displacement: *H* = 17.5, *p* = 0.35). Video recordings also showed that no physical displacement of the larvae occurred as a result of the impact of the pressure wave caused by the stimulus, in line with the minute physical characteristics of LDs. Notably, reaction probability increased with stimulus strength (Figure 2B, One-Way ANOVA, *F*_(2,69)_ = 40.8, *p* < 0.0001). Further, reacting larvae appeared to move towards the stimulus source (Figures 2C,D). We then measured the direction of their post-LD displacements (Figure 2E), and compared the proportions of displacements directed either towards or away from the source. We found that single LDs elicited left and right ~60° turns (relative to the body axis), as well as more frequent turns towards the stimulus source (Figure 2E, Rayleigh tests, towards: *Z* = 28.2, *p* < 0.0001, *μ* = 59.7°, *r* = 0.9, circular varianc*e* = 0.1°, *N* = 32, away: *Z* = 16.3, *p* < 0.0001, *μ* = 59.9°, *r* = 0.8, circular variance = 0.2°, *N* = 17, Pb_towards_ = 0.65, Two-tail Binomial test, *p* = 0.04). To confirm the non-stressful nature of LD-borne flows, we measured whole-body cortisol, a major stress hormone in teleosts (Wendelaar Bonga, 1997), in larvae previously exposed to LDs for 240 s. The results showed that LD-exposed and control larvae had similar levels of whole-body cortisol (Figure 2F, One sample *t*-test against 0, *t*_(9)_ = 0.1, *p* = 0.93). From these observations, we concluded that non-stressful water motions can elicit approach reactions in freely swimming larvae.

### Low-frequency LD-borne Flows Elicit Positive Taxis and Motion Damping

To further investigate correlates of LD-borne flows, we presented larvae with repetitive LDs, using two different protocols (Figure 3). In the first protocol (Figure 3A), LD onset was invariant to a larva’s position within the chamber, i.e., 1 ms LDs occurred at 1 Hz for 120 s (top panel). With this protocol, we observed that LDs caused larvae to shorten their distance to the stimulus source, also reducing their locomotor activity (Figure 3A, middle and bottom panels, respectively). Notably, they reached a state of almost complete immobility, and remained virtually immobile in the proximity of the source as long as LDs persisted. After LD offset, they recovered pre-stimulation locomotion levels only gradually. The larvae’s response to repetitive LDs, i.e., initial approach followed by sustained motion damping, thus appeared to involve teleonomic elements. To confirm this, we exposed them to a second protocol (Figure 3B) in which LD stimulation depended on a larva’s position within the chamber, i.e., 1 ms LDs occurred at 1 Hz only when and as long as the larva swam near the stimulus source (top panel). Again, we observed that LDs caused larvae to approach the source and reduce their locomotor activity (Figure 3B, middle and bottom panels, respectively). From these observations, we concluded that locally evoked water motions can elicit positive taxis and locomotion damping in freely swimming larvae.

### Motion Damping Depends on Distinctive Stimulus Properties and Sensory Background

We then set up to determine how distinct LD variables related to the magnitude of LD-mediated motion damping. For this we used a slightly modified variant of the first protocol (i.e., 1 ms LDs occurring for 30 s) and “motion change” (in %) as an estimate of response magnitude, defined as [(m_1_−*m*_0_)/*m*_0_]*100, where m_0_ and m_1_ were the integrals of distance swam every 10 ms (for 30 s) prior to and during LDs. We found that the response magnitude depended on LD frequency, i.e., 5 Hz decreased locomotion, whereas 100, 200 and 500 Hz increased locomotion, as compared to pre-stimulation baseline levels (Figure 4A, One-Way ANOVA, *F*_(8,126)_ = 10.9, *p* < 0.0001, followed by *post hoc* comparisons, *p* < 0.05). One sample *t*-tests against 0 motion change (in %) confirmed that motion damping occurred in response to LDs delivered at 1 or 5 Hz, but not at 0.25, 0.5, 10, 50, 100, 200 or 500 Hz (Figure 4A, 0.25 Hz: *t*_(7)_ = 0.8, *p* = 0.43, 0.5 Hz: *t*_(7)_ = 0.8, *p* = 0.46, 1 Hz: *t*_(31)_ = 3.7, *p* = 0.0009, 5 Hz: *t*_(22)_ = 10.4, *p* < 0.0001, 10 Hz: *t*_(12)_ = 2.0, *p* = 0.06, 50 Hz: *t*_(9)_ = 1.0, *p* = 0.37, 100 Hz: *t*_(10)_ = 2.1, *p* = 0.06, 200 Hz: *t*_(10)_ = 2.2, *p* = 0.06, and 500 Hz: *t*_(10)_ = 1.5, *p* = 0.17). Between 1 and 5 Hz, 5 Hz caused a larger motion reduction than 1 Hz (Figure 4A, Two-tailed *t*-test, *t*_(53)_ = 4.2, *p* = 0.0001). Therefore, we next examined the relation between LD speed and response magnitude using 1 ms LDs delivered at 1 Hz, in order to avoid possible ceiling effects. We found that response magnitude increased together with LD speed, i.e., input voltage (Figure 4B, Kruskal-Wallis test, *H* = 6.4, *p* = 0.04, followed by Dunn’s multiple comparisons, *p* < 0.05, linear regression, *F*_(1,15)_ = 7.5, *p* = 0.02). Next, also using a stimulus frequency of 1 Hz, we tested the effect of delivering either single LDs or, instead, trains of either 10 or 100 LDs on the magnitude of the motor response; trains of LDs were applied with an inter-LD interval of 1 ms, i.e., with a within-train frequency of 500 Hz. The results showed that LD-mediated motion change did not differ across these groups (Figure 4C, One-Way ANOVA, *F*_(2,37)_ = 1.0, *p* = 0.38), although results from One sample *t*-tests against 0 motion change (in %) indicated that trains of 10 LDs of minimum stimulus strength (0.1 V) were sufficient to damp locomotion (Figure 4C, One sample *t*-test against 0, 1 LD: *t*_(13)_ = 1.5, *p* = 0.17, 10 LDs: *t*_(9)_ = 2.3, *p* = 0.04, 99 LDs: *t*_(13)_ = 3.4, *p* = 0.005). To survey the effect of sensory background on response magnitude, we compared responses from larvae exposed to LDs (i.e., trains of 10 LDs of maximum strength 1 V delivered at 1 Hz for 120 s) against backgrounds of either stagnant or flowing medium (flow: 200 μl*min^−1^). Although still present (One sample *t*-test against 0, w/o flow: *t*_(19)_ = 17.8, *p* < 0.0001, with flow: *t*_(40)_ = 9.3, *p* < 0.0001), motion damping had reduced magnitude against a background of flowing medium, i.e., under decreased signal-to-noise ratio conditions (Figure 4D, Two-tailed *t*-test, *t*_(59)_ = 4.1, *p* = 0.0001). From these results, we concluded that the magnitude of LD-mediated motion damping depends on stimulus frequency, stimulus strength and sensory background.

### The Response Depends on Rearing Conditions and Can be Elicited Early in Development

Surprisingly, we found that larvae that had been raised individually prior to testing, i.e., deprived from hydrodynamic stimuli from swimming conspecifics, had a greater response magnitude than larvae that had been raised in groups (Figure 4E, Two-tailed *t*-test, *t*_(26)_ = 7.5, *p* < 0.0001; One sample *t*-test against 0, in groups: *t*_(13)_ = 4.8, *p* = 0.0003, individually: *t*_(13)_ = 17.2, *p* < 0.0001). Also, we observed that LDs elicited motion damping at 4 dpf already (One sample *t*-test against 0, *t*_(14)_ = 5.1, *p* = 0.0002, using 10 LDs of 0.1 V at 1 Hz, not shown). Further, from 4 to 6 dpf, the magnitude of motion damping remained invariant to age (One-Way ANOVA, *F*_(2,49)_ = 0.3, *p* = 0.73, not shown).

### LD-borne Flows Increase Threshold Levels of Response to Parallel Input

Larvae being exposed to repetitive LDs were less likely to respond to a second, parallel input (Figure 5). Transient hyperosmolarity is a potent stressful stimulus that causes avoidance reactions, increased locomotion and elevated whole-body cortisol (De Marco et al., 2014). However, it failed to alter the state of almost complete immobility caused by repetitive LDs (Figures 5A,B, Two-Way ANOVA, Stimulation factor: *F*_(1,36)_ = 125.5, *p* < 0.0001, NaCl concentration factor: *F*_(1,36)_ = 20.1, *p* < 0.0001, Stimulation × NaCl concentration factor: *F*_(1,36)_ = 1.1, *p* = 0.31). A square pulse of flashing blue light can also act as a potent stimulus, causing a brief locomotion increase after stimulus onset (Figure 5C); if prolonged, it leads to reduced locomotion and elevated whole-body cortisol (De Marco et al., 2013). However, a square pulse of flashing blue light that generally increases locomotion failed to alter the already reduced locomotor activity of LD-exposed larvae (Figure 5D, Mann-Whitney, *p* < 0.0001; Wilcoxon Signed Rank test against 0, without LDs: *p* = 0.002, with LDs: *p* = 0.82). From these results, we concluded that exposure to repetitive LDs can increase threshold levels of response to parallel input.

### Locomotion During and After LDs

**Figure 6:**
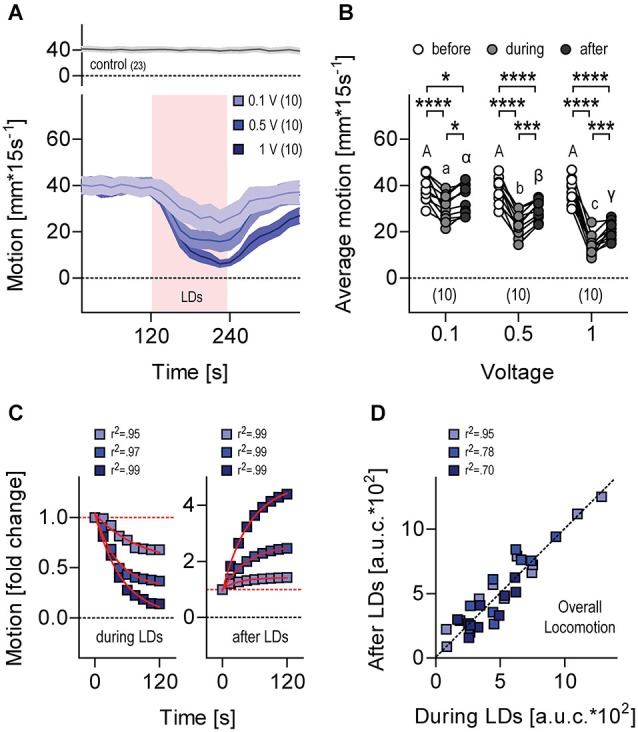
**Locomotion during and after LDs. (A)** Motion level (in mm*(15 s)^−1^) by single larvae before, during (light red background) and after LDs. Stimulation, trains of 10 LDs (500 Hz) delivered at 1 Hz for 120 s. Stimulus strength, 0.1 V (violet), 0.5 V (light blue) and 1 V (dark blue). Gray trace on top corresponds to data gathered in the absence of LDs. Lines and colored areas indicate means ± S.E.M. for each group, respectively. Sample size in parentheses. **(B)** Average motion from **(A)** before, during and after LDs, as a function of stimulus strength. Letters and asterisks above data points indicate results from *post hoc* comparisons after a Two-Way Repeated Measures ANOVA. Sample size in parentheses. **(C)** Normalized motion from **(A)** after the onset (left) and offset (right) of LDs. Fold change was calculated as F.C. = mm*(15 s)^−1^ at any given time interval / mm*(15 s)^−1^ immediately before the onset (left) or the offset (right) of LDs. Red lines indicate exponential fits. **(D)** Overall locomotion from **(A)** during and after LDs, calculated for each larva as the integral (area under the curve, a.u.c.) of the distance swum every 15 s over a time period of 120 s, following the onset and offset of LDs, respectively.

Larvae swimming in darkness at constant temperature showed regular locomotion (Figure 6A, top). When exposed to the first protocol of repetitive (1 Hz) LDs (Figure 3A), their level of locomotion decreased and increased after LD onset and LD offset, respectively (Figure 6A, bottom), with locomotion depending on stimulus strength, i.e., LD speed, as determined by the input voltage applied to the piezo actuator. As a result, the higher the strength of the stimulus the lower the overall level of locomotion recorded over 120 s, both during and after LDs (Figure 6B, Two-Way Repeated Measures ANOVA, Time factor: *F*_(2,54)_ = 173.1, *p* < 0.0001, Voltage factor: *F*_(2,27)_ = 12.8, *p* = 0.0001, Time × Voltage factor: *F*_(4,54)_ = 15.9, *p* < 0.0001, followed by *post hoc* comparisons). Exponential fits of the data confirmed that locomotion changed in a stimulus-strength-dependent manner only gradually after the onset and the offset of LDs (Figure 6C, decrease during LDs: 0.1 V, *r*^2^ = 0.95, 0.5 V, *r*^2^ = 0.97, 1 V, *r*^2^ = 0.99, increase after LDs, 0.1 V, *r*^2^ = 0.99, 0.5 V, *r*^2^ = 0.99, 1 V, *r*^2^ = 0.99). Also, the overall level of locomotion during LDs correlated well with the overall level of locomotion after LDs (Figure 6D, Pearson’s correlation, 0.1 V: *R*^2^ = 0.95, *p* < 0.0001, *N* = 10, 0.5 V: *R*^2^ = 0.78, *p* = 0.0007, *N* = 10, 1 V: *R*^2^ = 0.70, *p* = 0.005, *N* = 9).

### The Response Remains Stable Over Multiple Stimulations

**Figure 7:**
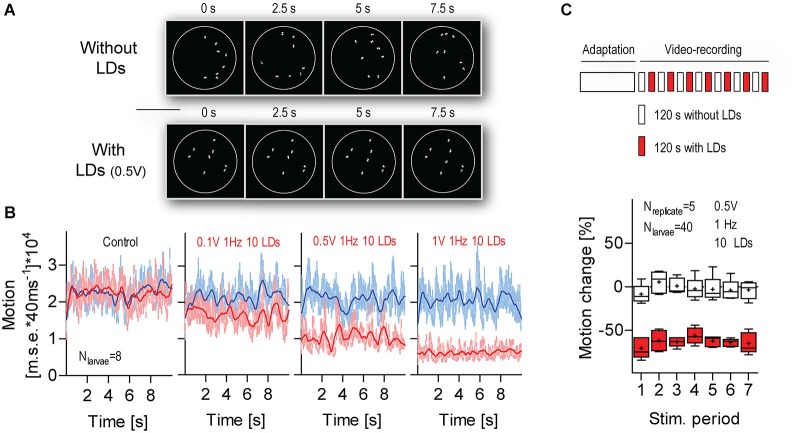
**The response to LD-borne flows remains stable over multiple stimulations. (A)** Exemplary images of sequences of transformed video frames (every 2.5 s) used to quantify the overall motion of a group of freely swimming larvae within the chamber (white circle), in combination with LD stimulation. Top: without LDs, bottom: with LDs. Note that only a larva’s eyes appear as white objects on a black background. Scale bar, 10 mm. See also Section Materials and Methods. **(B)** Motion level as calculated by a tracking algorithm that computes the pixel-by-pixel *m.s.e*. between transformed images, as shown in **(A)**, from consecutive video frames. Frame rate: 25 frames*s^−1^. Blue and red traces correspond to data gathered in time periods 1 (10 s before LD onset) and 2 (110 s after LD onset), respectively. In “control” (left), LDs did not occur during time period 2. Lines denote smoothed average values. **(C)** Top: Schematics of the multiple stimulation protocol. Bottom: Box-and-whisker plot of motion change (in %) in the absence (white boxes) or presence (red boxes) of LDs. Whiskers: min. to max., line: median, crosses: means. Shown are LD variables. See also Section Materials and Results.

Next, we assessed variations in response magnitude over multiple LD stimulations (Figure 7). For this, we exposed groups of larvae to a series of consecutive 120 s stimulation periods (applied every 240 s). In order to measure the overall motion of groups of swimming larvae, we used a tracking algorithm that computes the pixel-by-pixel *m.s.e*. between transformed images from consecutive video frames (Figure 7A, top, see also Section Materials and Methods). For each group, we calculated the area under the curve (a.u.c.) from ensued *m.s.e*. values (in 10 s periods) recorded immediately before (period 1, P1) and 110 s after LD onset (period 2, P2) (Figure 7B, bottom). Next, we calculated a global measure of “motion change”, as the difference (in %) between a.u.c. values from P1 and P2, or [(P2_a.u.c._–P1_a.u.c._)/P2_a.u.c._]*100. The results showed that larvae reduced their level of locomotion consistently over the several LD stimulations (Figure 7C, Two-Way Repeated Measures ANOVA, Stimulation factor: *F*_(1,48)_ = 198.5, *p* < 0.0001, Time factor: *F*_(6,48)_ = 2.0, *p* = 0.09, Stimulation × Time factor: *F*_(6,48)_ = 0.6, *p* = 0.71). We concluded that the larvae’s motion damping response to LD-borne flows remains stable over multiple stimulations.

### Lateral Line Dependance

**Figure 8:**
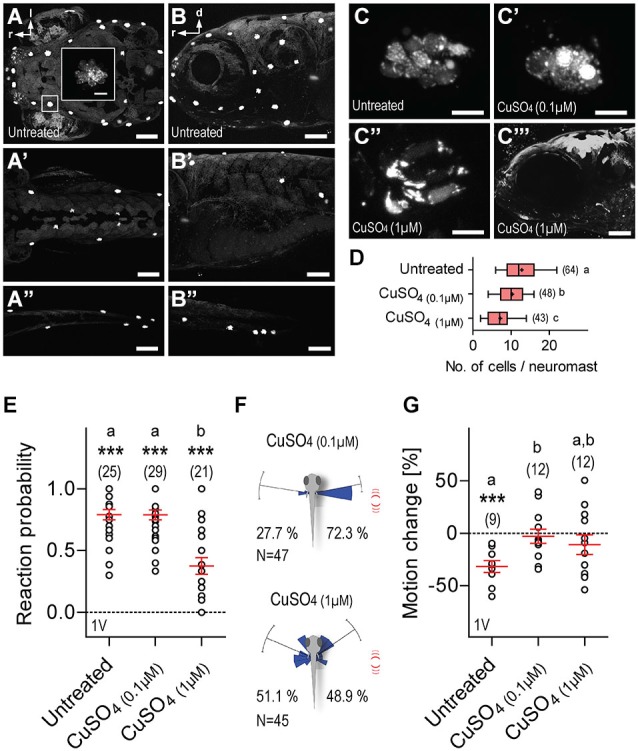
**Lateral line dependance. (A–B”)** NeuroTrace staining showing neuromasts in untreated (control) larvae as seen in dorsal **(A–A”)** and lateral **(B–B”)** views of the head **(A,B)**, trunk **(A’,B’)**, and tail **(A”,B”). (C–C”)** Stained hair cells as they appear in neuromasts of untreated larvae **(C)**, or after treatment with 0.1 μM **(C’)**, or 1 μM **(C”)** CuSO_4_. **(C”’)** Lateral view of the head after treatment with 1 μM CuSO_4_. **(D)** Cell numbers per neuromast as counted in images of the head. **(E)** Reaction probability as in Figure 2B. **(F)** Direction of movement relative to body axis (0°) upon a single 1 ms LD (as in Figure 2E) in CuSO_4_-treated larvae. Stimulus source (red lines) at 60–120°. **(G)** LD-induced motion change (in %, relative to pre-stimulation level) in untreated and CuSO_4_-treated larvae. **(D,E,G)** Sample size in parentheses. Letters indicate results from *post hoc* comparisons after One-Way ANOVAs, *p* < 0.05. **(D,E,G)** Asterisks over data groups indicate results from One sample *t*-tests against 0, ***p < 0.001. Scale bars, 100 μm **(A–B”,C”’)**, 10 μm (inset in **A**,**C–C”**).

Lastly, we examined the contribution of the lateral line to LD-mediated approach reactions and motion damping. For this, 5 dpf larvae were incubated overnight in either 0.1 or 1 μM CuSO_4_, a potent ototoxin promoting hair cell death in lateral line neuromasts, but not in the inner ear (Hernández et al., 2006; Olivari et al., 2008; Buck et al., 2012). We confirmed hair cell ablation in CuSO_4_-treated larvae via NeuroTrace staining immediately after incubation. Hair cells were labeled by NeuroTrace in untreated larvae (Figures 8A–C), and CuSO_4_-treated larvae showed on average a 0.7-fold reduction in the number of neuromasts in the head, trunk and tail, as compared to untreated larvae (fold reduction, CuSO_4(0.1 μM)_: 0.66, *N* = 6, CuSO_4(1 μM)_: 0.77, *N* = 6). Incubation in CuSO_4_ caused a dose-dependent disintegration of neuromasts (Figures 8C’–C”’), and reduced number of hair cells per neuromast (Figure 8D, One-Way ANOVA, *F*_(2,154)_ = 25.9, *p* < 0.0001 followed by *post hoc* comparisons, *p* < 0.05). We found that single LDs elicited reactions in all three groups of larvae, untreated, CuSO_4(0.1 μM)_ and CuSO_4(1 μM)_ (Figure 8E, Wilcoxon Signed Rank test against 0, untreated: *p* < 0.0001, CuSO_4(0.1 μM)_: *p* = 0.0003, CuSO_4(1 μM)_:* p* = 0.0002). Untreated and CuSO_4(0.1 μM)_-treated larvae were equally likely to react to single (1 ms) LDs, whereas larvae pre-incubated in CuSO_4(1 μM)_ were less reactive than untreated and CuSO_4(0.1 μM)_-treated larvae (Figure 8E, Kruskal-Wallis test, *H* = 24.5, *p* < 0.0001, followed by Dunn’s multiple comparisons, *p* < 0.001). Also, single LDs elicited more frequent turns towards the stimulus source in CuSO_4(0.1 μM)_-treated larvae (Figure 8F, top, Rayleigh tests, towards: *Z* = 27.4, *p* < 0.0001, *μ* = 79.6°, *r* = 0.9, circular variance = 0.1°, *N* = 34, away: *Z* = 10.7, *p* < 0.0001, μ = 80.8°, *r* = 0.9, circular variance = 0.1°, *N* = 13, Pb_towards_ = 0.72, Two-tail Binomial test, *p* = 0.001), but not in CuSO_4(1 μM)_-treated larvae (Figure 8F, bottom, Rayleigh tests, towards: *Z* = 15.3, *p* < 0.0001, μ = 55.3°, *r* = 0.8, circular variance = 0.2°, *N* = 22, away: *Z* = 13.9, *p* < 0.0001, μ = 66.7°, *r* = 0.8, circular variance = 0.2°, *N* = 23, Pb_towards_ = 0.49, Two-tail Binomial test, *p* = 1.0). Notably, both groups of CuSO_4_-treated larvae failed to show reduced locomotion after LD onset (Figure 8G, One-Way ANOVA, *F*_(2,32)_ = 3.5, *p* < 0.05, followed by *post hoc* comparisons, *p* < 0.05, One sample *t*-test against 0, untreated: *t*_(8)_ = 5.6, *p* = 0.0005, CuSO_4(0.1 μM)_: *t*_(11)_ = 0.4, *p* = 0.68, CuSO_4(1 μM)_:* t*_(11)_ = 1.2, *p* = 0.27). The latter result could not be accounted for by CuSO_4_-mediated hypertaxia, as similar levels of baseline locomotion, i.e., average distance swum every 15 s, measured over 120 s, were recorded in both untreated and CuSO_4_-treated larvae 10 minutes after incubation, (One-Way ANOVA, *F*_(2,29)_ = 0.44, *p* = 0.65; untreated: 49.1 ± 3.7 mm*(15 s)^−1^, CuSO_4(0.1 μM)_: 48.7 ± 3.2 mm*(15 s)^−1^, CuSO_4(1 μM)_: 44.9 ± 4.1 mm*(15 s)^−1^). From these observations, we concluded that a zebrafish larva’s response to LDs depends on the integrity of the lateral line.

## Discussion

In sum, we found that larval zebrafish execute approach reactions followed by a form of positive taxis and gradual motion damping in response to flows derived from local, non-stressful water motions. We also found that locomotion decreases and increases only gradually after the onset and offset of the stimuli, respectively. The motor response is highly stereotyped, depends on a narrow range of stimulus frequencies and encompasses increased threshold levels of response to parallel input. We show that response magnitude remains stable over multiple stimulations and depends on distinct stimulus properties, sensory background and rearing conditions. Also, hair cell ablation shows that the response depends on the lateral line, shown to be responsive to low-frequency stimuli (Coombs and Montgomery, 1994; Engelmann et al., 2000; Higgs and Radford, 2013).

Larval zebrafish have been shown to display rheotaxis (Olszewski et al., 2012; Suli et al., 2012) and escape reactions to motion and pressure waves (Kimmel et al., 1974; Zeddies and Fay, 2005; Burgess and Granato, 2007; Best et al., 2008; McHenry et al., 2009; Roberts et al., 2011; Kohashi et al., 2012; Olszewski et al., 2012; Bhandiwad et al., 2013; Stewart et al., 2013). Further, the analysis of acoustic startles (Kimmel et al., 1974) helped to reveal habituation (Best et al., 2008), prepulse inhibition (Burgess and Granato, 2007; Bhandiwad et al., 2013) and interactions between mechanosensory and visual pathways (Mu et al., 2012). Phonotaxis has also been observed in adult fish of several other species, as well as in coral reef larvae (Tolimieri et al., 2000, 2004). For example, it has been shown that round gobies (*Neogobius melanostomus*) respond to conspecific vocalizations (Rollo et al., 2007), and that gravid midshipman females (*Porichthys notatus*) approach sources of male calls and artificial tones (McKibben and Bass, 1998; Zeddies et al., 2010, 2012). Approach reactions to water motions are well documented too. Examples are studies of mottled sculpins (*Cottus bairdii*) (Braun and Coombs, 2000) and blind cave fish (*Astyanax mexicanus*) (Yoshizawa et al., 2010), shown to approach vibrating objects, and of striped panchax (*Aplocheilus lineatus*) and the African butterfly fish (*Pantodon buchholzi*), shown to orient according to the pattern of artificial prey-borne surface waves (Bleckmann and Schwartz, 1982; Hoin-Radkovsky et al., 1984). The results described here add a robust non-visual phenotype to a growing repertoire of laboratory behaviors in larval zebrafish. The question of what natural stimuli are being mimicked by LD-borne flows remains still open, as water motions of low and intermediate frequencies can arise from various sources. For example, flows from adult fish tend to be below 10 Hz (Bleckmann et al., 1991), isopods have a stroke cycle frequency of 6–10 Hz (Alexander, 1988), *Artemia* larvae show antennal beat frequencies of 6.7–9.5 Hz, depending on their developmental stage (Williams, 1994), and paramecia have a cilia beat frequency of 15–45 Hz (Funfak et al., 2015). Zebrafish larvae have tail beat frequencies of ~33 and 56–73 Hz during slow starts and cyclic swimming bouts, respectively (Müller and van Leeuwen, 2004), and cause vortices behind them lasting hundreds of milliseconds (Müller et al., 2000, 2008). Their pectoral fin movements occur at ~17 Hz in absence of axial body wakes (Green et al., 2011), and can be synchronized to the bending of the body (Thorsen et al., 2004). The response reported here comprises positive taxis, motion damping and sustained responsiveness to minute water jets. Presumably, joint action by these elements can increase detection of ethologically relevant stimuli by reducing distance-dependent attenuations of sub-threshold hydrodynamic fields as well as self-generated flows, thereby increasing signal-to-noise ratios of relevant inputs.

The lateral line comprises individual neuromasts distributed along the body, each containing groups of direction-selective hair cells (Webb, 2014). The directional selectivity of hair cells is morphologically grounded, i.e., displacement of the hair bundle toward or away from the kinocilium results in increased or decreased firing rate, respectively. Hence the firing rate of afferent fibers depends on stimulus strength as well as direction, and accurate stimulus decoding needs activation of a population of afferent fibers (Chagnaud and Coombs, 2014). The functional overlap between the lateral line and the inner ear has yet to be fully specified (Bleckmann, 1986; Hawkins, 1986; Braun et al., 2002; Braun and Sand, 2014). Generally, separate pathways of lateral line and auditory information processing exist, and perceptual interactions are thought to occur in the hind- and forebrain (Braun and Sand, 2014). In zebrafish, the lateral line gathers inhibitory feedback and excitatory modulation from the hind- and forebrain, respectively (Ghysen and Dambly-Chaudière, 2004). Efferent inputs appear to decrease self-motion-mediated hair cell activity (Chagnaud and Coombs, 2014), although reduced locomotion may enhance detection of relevant inputs—in line with this, motionless zebrafish larvae are more likely to escape from threatening flows, as compared to swimming larvae (Feitl et al., 2010). Hair cell ablation has been shown to alter acoustic startles in goldfish (*Carassius auratus*) and cichlids (e.g., *Haplochromis burtoni*) (Canfield and Rose, 1996; Mirjany et al., 2011), approach reactions in sculpins (Braun and Coombs, 2000), flow detection in blind cave fish (Baker and Montgomery, 1999), and rheotaxis (Olszewski et al., 2012; Suli et al., 2012) and escape reactions in larval zebrafish (Olszewski et al., 2012). Here, it altered the directional bias of the larvae’s reactions to single LDs, as well as their motion damping response to repeated LDs. Taken together, these results provide an interesting opportunity for the assessment of lateral line function, used extensively in ototoxicity and hair cell regeneration studies (Coffin et al., 2014), and possibly also for analyses of signal interactions in the octavolateralis system.

Also importantly, individually raised larvae lacking experience with hydrodynamic stimuli from conspecifics showed a greater response magnitude than larvae raised in groups. Likewise, low-density raised zebrafish displayed increased startle sensitivity (Burgess and Granato, 2008; Buck et al., 2012). The deposition of neuromasts in larval zebrafish is thought to be an intrinsic process, unaltered by experimental interference (Ghysen and Dambly-Chaudière, 2004). As the body of teleosts grows, the lateral line expands by generating new neuromasts, with organogenesis occurring either by activation of quiescent stem cells between neighbor neuromasts or through budding forming from pre-existing neuromasts (Ghysen and Dambly-Chaudière, 2007). The number and distribution of neuromasts in adult fish can vary greatly among genetically identical individuals, as opposed to a fairly regular distribution of neuromasts at the end of embryonic development. This suggests that the bodily pattern of neuromasts across related species may reflect ecology (Wada et al., 2008). If combined with varying amounts of hydrodynamic stimulation during early development, the response described here may also help to identify correlates of environmentally driven phenotypic variability in the origin of secondary neuromasts. Further, altogether the results uncovered a response that meets four categories of processes widely used in animal research to identify attention-like phenomena (Bushnell, 1998): orienting, as unconditioned species-specific responses (Figures 2C–E, 3); stimulus differentiation, as selective responsiveness to stimuli and discrimination against sensory background (Figures 4A,D); parallel processing, as altered capacities to process parallel inputs (Figure 5); and sustained responsiveness, as the ability to respond to stimuli over prolonged periods of time (Figures 3, 6). This would prove fruitful for behavioral screens, given the growing use of larval zebrafish for analyses of arousal (Yokogawa et al., 2012; Woods et al., 2014), high-throughput genetics (Patton and Zon, 2001) and pharmacological screening (Lessman, 2011).

## Author Contributions

RDM and SR conceived the project. RDM designed the study. AG, UH and RDM performed experiments. AG and RDM analyzed data. AG and RDM drafted the manuscript. RDM wrote the manuscript with contributions from all other authors.

## Conflict of Interest Statement

International patent No. WO2014/086938 describes some of the concepts presented in this manuscript.
